# Cold Atmospheric Plasma Is a Promising Alternative Treatment Option in Case of Split Skin Graft Failure

**DOI:** 10.1155/2024/1013445

**Published:** 2024-04-03

**Authors:** Aydar Khabipov, Andre Schreiber, Stephan Kersting, Richard Hummel, Johannes Höhn, Lars-Ivo Partecke, Sander Bekeschus, Anne Glitsch, Wolfram Keßler

**Affiliations:** ^1^Department of General, Visceral, Thoracic, and Vascular Surgery, Greifswald University Medical Center, Greifswald, Germany; ^2^Department of General, Visceral, and Thoracic Surgery, Helios Clinic Schleswig, Schleswig, Germany; ^3^ZIK plasmatis, Leibniz Institute for Plasma Science and Technology (INP), Greifswald, Germany; ^4^Clinic and Polyclinic for Dermatology and Venereology, Rostock University Medical Center, Rostock, Germany

## Abstract

Cold atmospheric plasma (CAP) has shown promising potential in promoting wound healing. This case report presents the successful application of CAP in a 42-year-old female patient with extensive wound healing disorders and superinfections following the excision of an abscess in the left thoracic region. After several failed split skin graft attempts, the implementation of CAP led to significant improvements in wound healing. This report highlights the wound healing-promoting effects of CAP and discusses its potential mechanisms of action.

## 1. Introduction

Wound healing disorders and superinfections pose significant challenges in clinical practice. Traditional treatment modalities often fail to achieve optimal outcomes. Cold atmospheric plasma is an ionized gas and can be described as the fourth matter of state. In the last decade, plasma medicine has been established and clinically approved, frequently found to possess antimicrobial properties and stimulate wound healing [1–3]. The mechanisms underlying its therapeutic effects include the generation of reactive oxygen species, which support eradicating the pathogen microbiome [4, 5] and are efficient against *Pseudomonas* bacteria [6–8]. Moreover, CAP can activate intrinsic wound healing mechanisms by inducing the migration and adhesion of molecules in skin cells [9], promoting VEGF secretion in skin cells [10, 11], and improving microcirculation in wounds [12, 13]. This case report illustrates the successful utilization of CAP in a challenging wound healing scenario, where conventional treatment modalities had failed.

## 2. Case Presentation

**Figure 1:**
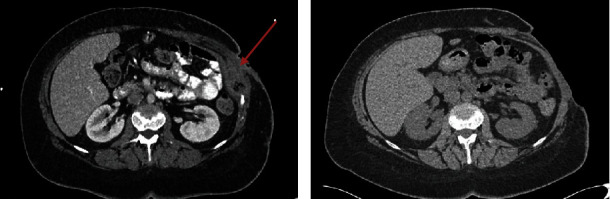
Computed tomography of abdomen (a) pre- and (b) postoperative. (a) Arrow marks the abscess with trapped air. (b) Postoperative lesion of the left abdominal wall.

A 42-year-old female patient with a history of nicotine abuse presented with extensive wound healing disorders (covering an area of 12 × 19 cm) and recurrent superinfections following the excision of a 5 cm subcutaneous abscess on the left thoracic region (Figure 1(a), red arrow marks the abscess). The initial abscess formed around a trocar incision site after laparoscopic ovarian cyst resection and was treated by incision and drainage months before. Wound healing was delayed, and healing disorders persisted since. A failed attempt at split skin grafting necessitated patient transfer from a smaller clinic to our university hospital for further management. A computed tomography (CT) scan revealed subcutaneous spread of abscesses. After surgical debridement of infected tissue on the left side of the thorax including the eleventh and twelfth rib, antibiotic therapy followed. Despite persisting wound area with low healing tendency and an express patient's wish to perform another split skin graft to close the wound, we rejected another attempt initially in respect to wound cultures positive for Pseudomonas aeruginosa and E. coli. Subsequent vacuum-assisted closure (VAC) and intraoperative rinsing were performed until negative wound cultures were achieved three times in a row. However, a second split skin graft failed. After additional VAC therapy and intensified wound care, a switch to a regimen of daily disinfections with polyhexanide was made. Noninflamed wound conditions and negative wound cultures were reached. In consideration of the patient's desire, the stable wound conditions, and a lack of options, a third attempt was made and seemed to be successful. However, the graft became infected after two weeks and had to be removed. The patient experienced chronic wound pain and required high doses of analgesics. Despite three weeks of conventional wound treatment carried out by specialized wound care nurses including antiseptic rinsing and daily change of dressings as well as application of topical antibiotics, the wound remained inflamed, reinfected with Pseudomonas aeruginosa, and showed no granulation tendency. Finally, cold atmospheric plasma therapy was initiated.

## 3. Treatment and Outcome

**Figure 2:**
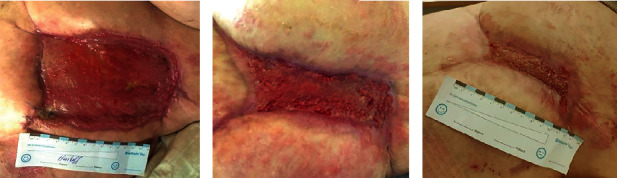
(a) Conventionally treated wound three weeks after failure of split skin graft, (b) wound after three weeks of CAP therapy, and (c) closed wound six weeks after start of CAP therapy.

Cold atmospheric plasma therapy was administered three times a week for a period of four weeks using the “kINPen MED” (neoplas med GmbH, Greifswald, Germany), a cold atmospheric pressure argon plasma jet device operating at an excitation frequency of roughly 1 MHz and generating a nominal output power of around 1 watt. The jet flow was 5 L/min and the wound area was treated for 3 s/cm^2^. The physical, electrical, and chemical specifications of the device have been extensively documented previously [14]. An antiseptic polyhexanide rinse was applied intermittently. Throughout the treatment course, wound cultures gradually became negative, bacterial biofilm and macroscopic signs of inflammation diminished, and the wound exhibited favorable granulation tendencies (Figure 2). The patient's pain levels decreased significantly and her psychological state improved. Weekly wound assessments were conducted. Wound closure was achieved six weeks after initiation of CAP therapy, allowing for significant reduction of analgesic usage. Regular follow-up examinations in our clinic showed no evidence of new infections or wound healing disorders.

## 4. Discussion

Wound infections are known as a crucial risk factor for wound healing disorders that can also appear during skin grafting [15–17]. The success of split skin grafting depends, among other factors, on the presence of *Pseudomonas aeruginosa* [18]. In view of stagnating wound conditions despite extensive conventional therapies and prompt improvement regarding wound infection and inflammation, it seems that CAP treatment induced wound healing in this challenging case. While the wound healing properties of CAP are widely recognized, it is noteworthy that this is the first reported case of CAP application following the failure of a split skin graft, to the best of our knowledge. Notably, CAP eradicated the persisting biofilm containing *Pseudomonas aeruginosa.* CAP generates a range of active oxygen and nitrogen products, including O, OH, O^2−^, NO, and H_2_O_2_. These reactive oxygen and nitrogen species (RONS) are capable to eliminate Pseudomonas aeruginosa via oxidative stress [4, 5, 8]. Moreover, CAP operates synergically with antibiotics [7], which could have played a beneficial role in the eradication of the bacterial load in this case. CAP reduced inflammation and facilitated granulation tissue formation as well, which have been reported before [2, 3]. Chronic inflammation is associated with elevated levels of interleukins and cytokines, which hinder wound closure [19]. CAP has the potential to regulate the inflammatory response by reducing cytokine production and facilitates the release of anti-inflammatory mediators like IL-10, TGF*β*, and IL-8 [20]. The patient experienced significant pain relief, leading to improved quality of life. The treatment of chronic wounds that are resistant to therapy over a long period of time is expensive and can sometimes take years. Consequently, investing in short-term cold plasma treatment may be a financially more advantageous option in the long term. This can lead to cost savings for the healthcare system, especially when using high-precision devices such as kINPen MED. For this reason, the central German Healthcare Board (G-BA) is currently preparing a comparative clinical trial to assess the benefits of including medical gas plasma treatment into the standard of care catalogue for wound treatment. Furthermore, it is conceivable that applying CAP to the wound before a split skin graft could eliminate bacteria and enhance the success rate of the skin graft. To prove this thesis, clinical studies are needed. A limitation of this case report is the lack of knowledge about potential synergies between polyhexanide and CAP, making it difficult to assess the efficacy of CAP in isolation. However, it is worth noting that prior treatment with antiseptic rinses and antibiotics alone had proven to be insufficient.

## 5. Conclusion

This case report illustrates the successful implementation of cold atmospheric plasma therapy as a sufficient method to eradicate Pseudomonas aeruginosa and promote wound healing after multiple failures of split skin graft in a complex surgical case. Further research illuminating synergy of CAP with antibiotics and antiseptic rinses in infections and larger-scale studies is warranted to explore the full potential of CAP in diverse clinical scenarios.

## Data Availability

CT images and pictures of wound development are extracted from IT System “Meyerhofer” of University Hospital of Greifswald and can be requested with exact dates. Laboratory parameters representing inflammation could be supplemented. In case of request, please correspond to aydar.khabipov@med.uni-greifswald.de.
